# Transcriptional and Posttranscriptional Regulations of the *HLA-G* Gene

**DOI:** 10.1155/2014/734068

**Published:** 2014-03-13

**Authors:** Erick C. Castelli, Luciana C. Veiga-Castelli, Layale Yaghi, Philippe Moreau, Eduardo A. Donadi

**Affiliations:** ^1^Departamento de Patologia, Faculdade de Medicina de Botucatu, Universidade Estadual Paulista (UNESP), 18618-970 Botucatu, SP, Brazil; ^2^Division of Clinical Immunology, Department of Medicine, School of Medicine of Ribeirao Preto, University of São Paulo (USP), 14049-900 Ribeirão Preto, SP, Brazil; ^3^Alternative Energies and Atomic Energy Commission, Institute of Emerging Diseases and Innovative Therapies, Department of Hematology and Immunology Research, Saint-Louis Hospital, 75010 Paris, France; ^4^Paris-Diderot University, Sorbonne Paris-Cité, UMR E5, University Institute of Hematology, Saint-Louis Hospital, 75010 Paris, France

## Abstract

*HLA-G* has a relevant role in immune response regulation. The overall structure of the *HLA-G* coding region has been maintained during the evolution process, in which most of its variable sites are synonymous mutations or coincide with introns, preserving major functional *HLA-G* properties. The *HLA-G* promoter region is different from the classical class I promoters, mainly because (i) it lacks regulatory responsive elements for IFN-**γ** and NF-*κ*B, (ii) the proximal promoter region (within 200 bases from the first translated ATG) does not mediate transactivation by the principal HLA class I transactivation mechanisms, and (iii) the presence of identified alternative regulatory elements (heat shock, progesterone and hypoxia-responsive elements) and unidentified responsive elements for IL-10, glucocorticoids, and other transcription factors is evident. At least three variable sites in the 3′ untranslated region have been studied that may influence *HLA-G* expression by modifying mRNA stability or microRNA binding sites, including the 14-base pair insertion/deletion, +3142C/G and +3187A/G polymorphisms. Other polymorphic sites have been described, but there are no functional studies on them. The *HLA-G* coding region polymorphisms might influence isoform production and at least two null alleles with premature stop codons have been described. We reviewed the structure of the *HLA-G* promoter region and its implication in transcriptional gene control, the structure of the *HLA-G* 3′UTR and the major actors of the posttranscriptional gene control, and, finally, the presence of regulatory elements in the coding region.

## 1. Introduction

The nonclassical HLA-G molecule presents several properties that differ from other classical class I HLA (-A, -B, and -C) molecules, including restricted tissue distribution; limited protein variability; presence of several membrane-bound and soluble isoforms; unique molecular structure, presenting a particular peptide-binding groove that impairs peptide presentation to T cells; ability to form dimers and polymers and a reduced cytoplasmic tail that impairs molecule turnover; and, most importantly, the molecule that modulates several functions of immune system cells (reviewed by [1]). The interaction of HLA-G with leukocyte receptors, particularly ILT-2 and ILT-4, downregulates the cytotoxic activity of T CD8 and Natural Killer cells and inhibits antigen presentation and lymphocyte proliferation [1, 2]. Dendritic cells expressing IL-10 and HLA-G can induce regulatory T cells [3]. Due to all of these properties, HLA-G has been recognized as a tolerogenic molecule, and the tissue expression of HLA-G may protect or harm; that is, it may protect allografts against attack by the recipient immune system and may impair the cytotoxic immune response against tumor cells.

The* HLA-G* gene also presents unique features. The coding region exhibits few polymorphic sites randomly distributed along exons and introns, contrasting with the high rate of exonic polymorphic sites observed in classical HLA class I genes. The exonic nucleotide sequences encoding residues that are important for molecule dimerization and molecule interaction with leucocyte receptors are usually conserved, indicating that the overall structure of the molecule was maintained throughout human evolution [1, 2, 4, 5]. Considering that HLA-G is expressed on the surface of placenta trophoblast cells, allowing the fetus to properly develop despite the maternal immune response, some sort of functional conservation was expected. On the other hand, gene regulatory regions present several polymorphic sites close to nucleotide sequences that serve as gene regulatory elements [6–9]. Nucleotide variability in the promoter region may influence HLA-G levels by modifying binding affinity for transcription factors. In contrast to classical HLA class I genes, the promoter region of* HLA-G *does not have responsive elements for IFN-*γ* or NF-*κ*B. Similarly, nucleotide variability at the 3′ untranslated region (3′UTR) may influence* HLA-G* mRNA stability, microRNA targeting, or both, affecting the posttranscriptional gene regulation.

Considering that the structure of HLA-G molecules has been maintained throughout evolution, the quantity of produced molecules may primarily depend on factors that modulate gene expression by transcriptional and posttranscriptional mechanisms. Firstly, we will review the structure of the* HLA-G* promoter region and its implication in transcriptional gene control; secondly, the structure of the* HLA-G* 3′UTR and the major actors of the posttranscriptional gene control; and, finally, the presence of elements in the coding region that may regulate gene expression and differential mRNA splicing.

There is no consensus regarding the positions of the nucleotide variation in the* HLA-G *promoter and 3′UTR, mainly because (i) the IMGT/HLA database only presents sequences within 300 bases upstream to the first translated ATG, (ii) the complete 3′UTR gene segment is not considered in the IMGT database, and (iii) several HLA alleles were described presenting only some exon sequences. Therefore, the nucleotide positions used in the present study follow the one presented in the NG_029039 sequence (http://www.ncbi.nlm.nih.gov/nuccore/NG_029039). The nucleotide named as +1 is the Adenine of the first translated ATG (position 5867 at NG_029039). Variations within regulatory elements in the upstream 5′ untranslated region and 5′ promoter were denoted as negative values, considering position 5866 at NG_029039 as nucleotide −1.

## 2. HLA-G Transcriptional Regulation

The HLA class I genes are usually very similar in nucleotide sequence and structure because most of these genes have been generated in a series of imperfect duplications [10]. Therefore, in general, the same regulatory elements are acting in HLA class I genes, with some differences for each HLA class I locus. The HLA class I promoters are usually conserved, presenting cis-acting regulatory elements mainly within 220 bases upstream to the first translated ATG. However, the* HLA-G* promoter is atypical compared to other HLA class I genes since most of these regulatory elements are not functional.

The* HLA-G* locus presents a tissue-restricted expression pattern, being expressed in physiological conditions only in certain tissues such as trophoblast at the maternal-fetal interface, thymus, cornea, pancreas, proximal nail matrix, erythroblast, and mesenchymal stem cells [1, 11–18]. In view of the immunomodulatory properties of the HLA-G molecule, its expression must be under a tight tissue-specific regulation.

**Figure 1:**
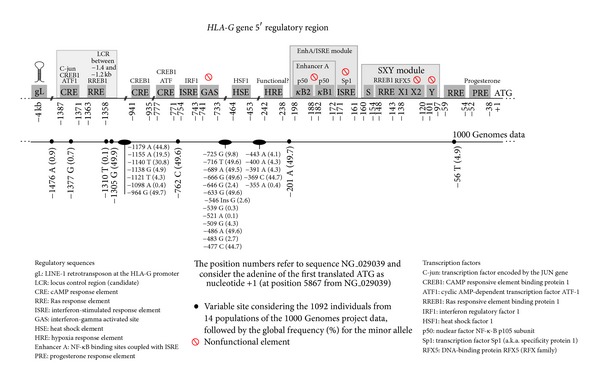
The* HLA-G* 5′ regulatory region with its known regulatory elements and variable sites according to the 1000 Genomes data.

Overall, HLA class I genes present two main regulatory modules in the proximal promoter region (200 bases upstream to the translation start point), including (i) the Enhancer A (EnhA) combined with an interferon-stimulated response element (ISRE) and (ii) the SXY module, in which the transcription apparatus is mounted (Figure 1) [19–24]. However, these regulatory elements present locus-specific differences leading to different levels of HLA class I constitutive- and induced-expression (reviewed at [24, 25]).

The EnhA element includes two adjacent palindromic NF-*κ*B binding sites (*κ*B1 and *κ*B2) that interact with the NF-*κ*B family of transcription factors, both important to the constitutive and/or induced expression of HLA class I genes. This family includes several members, such as p50, p65 (also known as RelA), p52, c-Rel, and RelB, all usually acting by forming homo- or heterodimers [19]. Theoretically, the interaction of these factors with the EnhA element may transactivate (acting on any *κ*B binding site) any HLA class I gene [20]. Thus, the HLA locus-specific transcription rate would be determined by (i) the levels of NF-*κ*B/Rel family proteins in different tissues, (ii) modifications in the regulatory sequences, and (iii) potential activation of different NF-*κ*B/Rel dimers [20]. In addition, EnhA may be a target sequence for other DNA-binding proteins, such as proteins of the leucine zipper transcription factor family [20]. For instance, p65 has a potent transactivation domain and might operate as a p65/p50 heterodimer or p65/p65 homodimer, while p50 lacks this transactivation domain and may not transactivate as a p50/p50 homodimer [20]. EnhA also mediates the TNF-induced transcription of HLA class I molecules [20, 29].

Due to variations in the EnhA nucleotide sequences among different HLA class I genes, NF-*κ*B/Rel factors may interact as homo- or heterodimers resulting in different transcription levels [20]. The* HLA-G* EnhA element (including *κ*B1 and *κ*B2 sites) encompasses nucleotides −198 and −172 (regarding NG_029039) and, compared to other HLA class I genes, it is the most divergent one [19, 20]. In fact, the *κ*B-sites in the* HLA-G* promoter (EnhA) are reported to bind only p50/p50 homodimers [25] (Figure 1). As presented earlier, p50 homodimers are not potent HLA class I gene transactivators [20]. Thus, although* HLA-G* possesses an NF-*κ*B responsive element, it is not as efficient as the HLA class I classical genes [25].

ISRE is a target site for the interferon regulatory factor family, including the interferon regulatory factor-1 (IRF-1, activator), IRF-2, and IRF-8 (inhibitors) [19]. Interferon-*γ* (IFN-*γ*) is the most potent cytokine inducing HLA class I gene expression. IFN-*γ* induces the expression of IRF-1 by the activation of the Janus kinases (jak) 1 and 2 and phosphorylation of Stat1 (JAK/STAT pathway) [19, 21]. ISRE is adjacent to the EnhA element (constituting the module EnhA/ISRE presented earlier) and, thus, ISRE and EnhA regulate HLA class I expression cooperatively (Figure 1). ISRE also participates in the transactivation of *β*2-microglobulin, which is associated with the heavy *α* chain of the HLA class I molecule [21], and this information is important because an unbalanced production of these chains may impair correct HLA molecule assembly.

The nucleotide sequence of ISRE also varies among HLA class I loci. In this respect, locus-specific differences were observed in the IFN-induced expression levels of HLA class I genes [21, 29–31]. The* HLA-A* locus, for instance, does not respond to IFN at the same level as* HLA-B* and* HLA-C*, probably because of differences in the ISRE structure [19, 21, 29–31]. However, when comparing ISRE of the* HLA-G* locus with other class I genes, encompassing nucleotides −171 to −161, the* HLA-G* gene presents the most divergent ISRE compared to the ISRE consensus sequence, followed by* HLA-E* [19, 21], raising the issue of whether or not this element is fully functional for* HLA-G* and* HLA-E*. In fact, neither* HLA-G* nor* HLA-E* ISREs mediate IFN-*γ*-induced transactivation, and the binding of IRF-1 is not detected for* HLA-G* [21]. However, in the same way, probably because of the defective nature of the* HLA-G* ISRE, the binding of IRF-2 (transcription repression) was also not detected for* HLA-G* [21].

ISRE is also a target for other protein complexes that may mediate HLA class I transactivation. However,* HLA-Gκ*B2 (EnhA) and ISRE seem to bind only the constitutively expressed factor Sp1 (also known as Specificity Protein 1) [21, 25]. Nevertheless, the binding of Sp1 does not modulate the constitutive or IFN-induced transactivation of HLA class I genes, including* HLA-G* [21]. On the other hand, a candidate interferon-gamma activated site (GAS) was described between the −741 and −733 positions, presenting a sequence that would be compatible with a GAS consensus sequence (Figure 1). However, besides this new candidate, IFN-*α*, IFN-*β*, and IFN-*γ* treatments failed to increase HLA-G expression, a fact that was accredited to the defective nature of both the new candidate and the EnhA/ISRE region [32–34]. Nonetheless, another study showed that IFN-*β* enhances HLA-G expression by another ISRE present next to the nonfunctional GAS element at positions −754 to −743 [34].

The SXY module comprises the S, X1, and X2 (also known as site *α*) boxes and the Y box (also known as the Enhancer B or CCAAT box). The X1 box is a target for the multiprotein complex regulatory factor X (RFX), including the RFX5, the regulatory factor X-associated protein (RFXAP), and RFXANK [20, 25, 35, 36]. These RFX members have been shown to interact with the class II transactivator (CIITA) [37, 38], which is also an important element for HLA class I gene transactivation [25]. The X2 box is a binding target for the ATF/CREB (Activating Transcription Factor/cAMP Response Element Binding protein) transcription factor family [39]. Box Y is a binding target for Nuclear Factor Y (NFY), including subunits alpha (NFYA), beta (NFYB), and gamma (NFYC) [25, 40]. The function of box S has not yet been elucidated. The binding of these factors to the SXY module allows the further binding of the coactivator CIITA and the NOD-like receptor family CARD domain containing 5 (NLRC5) factors [25, 41, 42]. The CIITA is constitutively expressed by antigen presenting cells and is induced by IFN-*γ*, and it transactivates HLA class I genes [41, 43]. NLRC5 transactivates HLA class I genes (but not HLA class II) and is constitutively expressed in a series of different tissues, mainly hematopoietic cells, or is induced by INF-*γ* [44–46].

For* HLA-G*, the SXY module presents sequences compatible only with the S and X1 elements, but divergent from X2 and Y elements (Figure 1) [25]. Therefore, CIITA, which is dependent on a functional SXY module, does not transactivate* HLA-G*, mainly because of the missing X2 and Y elements [25, 41, 42, 47, 48].

The* HLA-G* promoter region is unique among the HLA genes. Considering all the elements discussed above, it became clear that the* HLA-G* proximal promoter (within 200 bases) did not mediate transactivation by the principal HLA class I transactivation mechanisms [25]. In addition, studies evaluating the* HLA-G* promoter region within 1438 bp from ATG did not detect differences in the basal level of transactivation for different* HLA-G* promoters in different cell types [49, 50].

Some alternative regulatory elements within the* HLA-G* gene promoter have been described. A heat shock element (HSE) which would respond to the presence of heat shock proteins (HSP), especially the heat shock factor 1 (HSF1), was described in the* HLA-G* promoter region [51] (Figure 1). Stress-induced HSP are potent components that modulate immune responses. In general,* HLA-G *transcription is induced by heat shock (physical stress) or arsenate treatment (chemical stress) in human melanoma and glioblastoma cell lines, in which stress-induced HSF1 binds to an HSE lying between the −464 and −453 positions. This HSE response was detected for* HLA-G* but not for other HLA class I genes [51].

HLA-G expression may also be induced by progesterone [52], which is an immunomodulatory steroid hormone secreted both by the corpus luteum and placenta, allowing endometrium maintenance and embryo implantation. The mechanism underlying this induction is primarily mediated by the activation of the progesterone receptor (PR) and its subsequent binding to an alternative progesterone response element (PRE) found in the* HLA-G* promoter between positions −52 and −38, overlapping the* HLA-G* TATA box [53] (Figure 1).

Experiments with transgenic mice allowed the identification of a Locus Control Region (LCR) candidate located at least 1.2 kb upstream to the first translated ATG. This region is critical for the HLA-G expression regarding when and where it should be expressed. It is possible that this region acts by maintaining the chromatin in an open state or active configuration, enhancing gene expression [54, 55]. In addition, it may bind protein complexes associated with activation and inhibition of* HLA-G* transcription [56, 57].

At least three CRE/TRE candidate sites (cAMP Response Element/TPA Response Element) have been already considered, the first one being situated between the −1387 and −1371 positions (inside the putative LCR region discussed earlier), the second between the −941 and −935 positions, and the third between the −777 and −771 positions (Figure 1). The first CRE site (at LCR) was described to be an* in vitro* target site for c-jun by using electrophoretic mobility shift assay (EMSA). C-Jun, together with c-Fos, forms the AP-1 early response transcription factor. In addition, this same site was reported to bind ATF1/CREB1* in vitro* and* in situ* by using chromatin immunoprecipitation (ChIP) [39]. The second CRE/TRE site binds* in vitro* to CREB1 and the third site binds* in vitro* to ATF1/CREB1 [39]. Mutations in all three CRE/TRE sites have been reported to reduce the* HLA-G* CREB1 transactivation, but a stronger inhibition was observed when the first CRE/TRE site (at the LCR) was mutated [39].

The repressor factor RREB1 (Ras Responsive Element Binding 1) may also be implicated in* HLA-G* expression regulation. At least three binding sites for RREB1, known as Ras Response Elements (RRE), in the* HLA-G* promoter region have been described. The consensus sequence GGTCCT, corresponding to one of the binding sites for RREB1, was found in the proximal promoter between the −59 and −54 positions (one direct site) and between the −148 and −143 positions and the −139 and −134 positions (a direct site and an inverted site). A target site related to the other consensus-binding site for RREB1, CCCCACCATCCCC, was found within the LCR between the −1363 and −1358 positions (Figure 1). The mechanism underlying RREB1 repression is probably associated with the recruitment of the corepressor C-terminal binding protein 1 or 2 (CtBP-1 or CtBP-2), or both, and the deacetylase 1 (HDAC1), which is involved in chromatin remodeling probably increasing chromatin condensation and hampering transcription factor accessibility [58, 59].

The GLI-3 repressor, a signal transducer of the Hedgehog pathway (HH), has also been reported to regulate* HLA-G* during the maturation of osteoblasts [60], especially in the production of the HLA-G5 isoform. It acts by a direct interaction of the HH signaling transducer factor GLI-3 with the* HLA-G* promoter. However, it is not clear whether the HH signaling pathway, a highly conserved molecular pathway involved in the development of several tissues, directly regulates HLA-G5 expression in other cell types.

A negative regulator of gene expression is observed in a sequence about −4 Kb upstream to the* HLA-G* translation starting point, overlapping with a LINE-1 sequence [61] (Figure 1). LINEs (Long Interspersed Elements) are a group of retrotransposons, which are highly repetitive elements from the eukaryotic genome that contribute to genome variability. The LINE-1 element described for* HLA-G* (named gL) is an AT-rich sequence (about 60%) that presents more sites with a high probability of forming hairpin loops than the general LINE sequence. These hairpin loops might directly or indirectly interact with the* HLA-G* promoter and interfere with the binding of transcriptional factors and enhancers [61]. LINE elements are frequently found lying in the 5′ upstream regulatory region of other HLA class I genes, including* HLA-A*. However, the LINE sequence found in the* HLA-A* promoter (named aL) is not transcriptionally active and is shorter than the one found in* HLA-G* (gL). Therefore, the presence of this gL element in the* HLA-G* promoter would explain its limited expression compared with other class I genes. However, it should be noticed that this gL element is also present in HLA-G-expressing cells; thus, other regulatory elements might inhibit or overcome this negative regulation [61].

Hypoxia is an important physiological microenvironment for placentation and for the formation of the maternal-fetal interface [62]. The microenvironment is also crucial for the function of T and B cells. In this scenario, hypoxia is also associated with increased HLA-G expression. The Hypoxia-Inducible Factor (HIF) is involved in the control of cellular responses to oxygen depletion [62]. The HLA-G expression (membrane and soluble) is 2 times increased when extravillous cytotrophoblasts are cultivated under only 2% oxygen [63]. Likewise, hypoxia is associated with increased* HLA-G* transcription in a series of HLA-G-negative tumor lineages, such as 1074mel [64, 65] and M8 [66]. A consensus Hypoxia Response Element (HRE) [67] is located between the −242 and −238 positions (Figure 1). However, the functionality of this element has not been explored [64].

Some inducers of HLA-G expression have been described; but the underlying induction mechanisms are unknown. Interleukin 10 (IL-10), which is produced by lymphocytes, monocytes, macrophages, placenta, and some tumors, may induce HLA-G expression and the downregulation of other HLA class I and II genes [68–70]. Cortisol, a glucocorticoid produced by the adrenal gland, is a potent immunomodulatory hormone at high doses. HLA-G expression in trophoblastic cells was increased following treatment with dexamethasone or hydrocortisone [71], but no complete Glucocorticoid Response Element (GRE) has been identified in the* HLA-G* promoter.

Granulocyte-macrophage colony-stimulating factor (GM-CSF) is a protein secreted by macrophages, T cells, mast cells, NK cells, endothelial cells, fibroblasts, and uterine epithelium. GM-CSF increases HLA-G expression when combined with INF-*γ* treatment, but no effect is observed for GM-CSF alone [72, 73].

The Leukemia Inhibitory Factor (LIF) is a cytokine expressed at the maternal-fetal interface in the cytotrophoblast that plays an important role in implantation. LIF is mainly expressed in the implantation window. By using the choriocarcinoma cell line JEG3,* HLA-G* transcription was increased by about 3.6 times after LIF treatment. It was demonstrated that LIF induces full-length membrane HLA-G (HLA-G1) expression on the JEG3 cell surface [74]. In addition, LIF may induce HLA-G1 expression in the presence of ERAP1 (Endoplasmic Reticulum Aminopeptidase-1) expressed in the endoplasmic reticulum. Repression of ERAP1 in JEG3 cells treated with LIF diminishes HLA-G expression, suggesting a role for ERAP in* HLA-G* regulation [75].

Some drugs may also induce HLA-G production, such as methotrexate (MTX), one of the most used antirheumatic drugs for the treatment of rheumatoid arthritis (RA). MTX can induce apoptosis of mitogen-stimulated peripheral blood mononuclear cells (PBMCs) resembling the mechanisms underlying the inhibition of cytotoxic T CD8+ cell activity by soluble HLA-G molecules. MTX can induce the production of sHLA-G in unstimulated RA or healthy individual PBMCs and may have a role in the clinical outcome of RA patients. The mechanisms underlying sHLA-G production after MTX treatment are unknown, but it was reported that MTX therapy mediates an increase of interleukin-10-producing cells, which in turn may stimulate HLA-G production [76].

The* HLA-G* promoter exhibits numerous polymorphic sites (Figure 1). Data from the 1000 Genomes project, including 1092 individuals from 14 different populations, showed 32 variable sites within 1500 nucleotides upstream to the first translated ATG. Most of these variable sites have been already described in other populations or samples different from those evaluated by the 1000 Genomes consortium [6–9, 77–81]. Of those, 24 variable sites present frequencies higher than 1% and 14 present frequencies higher than 10% in the global 1000 Genomes data (all 1092 individuals). These variable sites may be important for the regulation of HLA-G expression and may act in different ways. Polymorphisms in the proximal promoter of* Paan-AG*, the functional homologue of* HLA-G* in the Olive Baboon, have been shown to influence NF-*κ*B binding and transcription activity [82, 83]. However, the human variable sites may act by mechanisms differing from those described above because, generally, these variable sites do not coincide with known regulatory elements (Figure 1).

Variation in regulatory elements may affect the binding of the corresponding regulatory factors. In this respect, only four variable sites coincide with known regulatory elements: (i) position −1377 in the first CRE site of the LCR, (ii) positions −1310 and −1305 of the LCR, and (iii) position −56 of the Ras Response Element (RRE) in the proximal promoter. Of these, only the ones at positions −1305 and −56 are frequently found worldwide (Figure 1). Other variable sites are close to known regulatory elements and may somehow influence the binding of transcription factors. In this group we may observe variable sites at positions −762 (between a CRE and ISRE), −725 (next to a nonfunctional GAS element), −477 and −433 (around the HSE), and −201 (next to Enhancer A) (Figure 1).

Few studies have associated promoter polymorphisms and differential HLA-G expression. The variable site at position −725, in which the minor allele (G) is present in 9.8% of the chromosomes evaluated in the 1000 Genomes project, was associated with differential HLA-G expression.* HLA-G* promoter haplotypes (between −1389 and −55 and not considering primer sequences) were cloned into luciferase expression vectors and transfected to the HLA-G expressing cell JEG-3, resulting in a significantly higher expression level of the promoters presenting Guanine at position −725 [84]. In addition, another study described the same influence of position −725 on HLA-G expression levels [85]. This same polymorphism (−725 G) has been reported to be associated with sporadic miscarriage [7] and end-stage renal disease [86], while the most frequent allele (−725 C) has been reported to protect against multiple sclerosis [87]. Nevertheless, despite the lack of studies regarding* HLA-G* promoter polymorphisms and HLA-G expression, some polymorphic sites have already been associated with several conditions. The polymorphism at position −964, which is very frequent among the populations evaluated by the 1000 Genomes consortium, was associated with asthma. The −964 G/G genotype was associated with asthmatic children of affected mothers, whereas the A/A genotype was associated with asthmatic children of unaffected mothers [88]. The −964 A and −486 C alleles, together with the −725 G allele, were also associated with protection against end-stage renal disease [86]. The polymorphism at position −1305, also very frequent among the 1000 Genomes populations, was associated with nonsegmental vitiligo [89].

The methylation status of the* HLA-G* promoter is also very important to the* HLA-G* transcriptional activity [90]. It has been reported that the CpG islands in the* HLA-G* promoter region of JAR (choriocarcinoma) cells, which does not express HLA-G, were fully methylated, whereas for an HLA-G expressing cell such as JEG-3, the CpG islands were only partially methylated [91, 92]. In addition, HLA-G expression was induced in several tumor cell lines by using demethylation agents, such as 5-aza-2′deoxycytidine [93–97]. Moreover, the levels of histone acetylation in the* HLA-G* promoter chromatin have been reported to be significantly enhanced in FON+ (melanoma) and JEG-3 (Human placental choriocarcinoma cell line) cell lines, both expressing HLA-G, while in non-HLA-G expressing cell lines, such as M8 (melanoma) and JAR, histones seem to be hypomethylated [94, 96, 98]. Histone acetylation is usually associated with a relaxed chromatin structure, therefore, with greater levels of gene expression [99, 100]. In this respect, polymorphisms in the* HLA-G* promoter, especially in the CpG islands, might also be associated with different methylation profiles [84].

**Figure 2:**
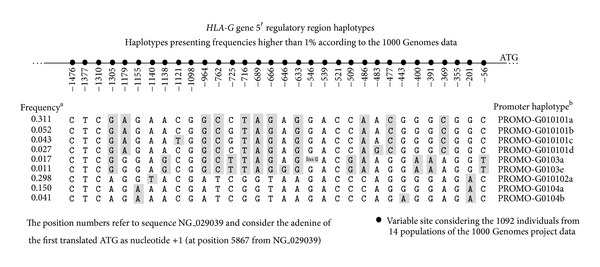
The most frequent haplotypes of the* HLA-G* 5′ regulatory region according to the 1000 Genomes data. ^a^Haplotype frequencies considering the 1092 individuals from the 1000 Genomes project [26]. ^b^Haplotypes were named according to [6].

Although most of the* HLA-G* promoter variable sites do not occur inside known regulatory elements (Figure 1), balancing selection has been reported to maintain divergent haplotypes in the 5′ promoter [6, 8, 9, 78, 79] and 3′UTR regulatory regions [6, 27, 28, 78, 101, 102]. In fact, at least 14 variable sites in the promoter region do present frequencies higher than 10%, and 11 variable sites present frequencies higher than 44% (Figure 1). However, considering the haplotypes described for the* HLA-G* promoter, which seem to be the same worldwide [6, 8, 9, 77–79], most of these frequent variable sites are in complete Linkage Disequilibrium (LD), and just four main* HLA-G* promoter lineages are associated with these variable sites. These promoter lineages were first proposed by Ober's group [8] and subsequently confirmed and named in a Brazilian study as PROMO-G010101, PROMO-G010102, PROMO-G0103, and PROMO-G0104 [6]. In addition, considering data from the 1000 Genomes Project, only nine promoter haplotypes present frequencies higher than 1% in worldwide populations (Figure 2), but two of them, PROMO-G010101a and PROMO-G010102a, which are the most divergent ones, account for more than 60% of all haplotypes. Nevertheless, despite the fact that most of these frequent* HLA-G* variable sites are not within known regulatory elements, several lines of evidence indicate balancing selection acting on the* HLA-G* promoter found in several populations [6, 8, 9, 78, 79], suggesting that divergent promoters have been maintained with high heterozygosis. This observation is probably related to a possible better fitness of individuals carrying both high- and low-expressing promoters. Therefore, these divergent* HLA-G* promoter haplotypes are probably associated with differential HLA-G expression, but the mechanisms are unknown. However, as discussed later, the pattern of LD observed for the promoter region extends up to* HLA-G* 3′UTR [6, 8, 9, 27, 77–79] and at least 20 kb beyond the* HLA-G* 3′UTR [102]. Thus, selective pressures acting on other* HLA-G* regions as well as adjacent sequences might also influence* HLA-G* promoter variability and heterozygosis. Figure 2 illustrates major* HLA-G *promoter region haplotypes observed in worldwide populations.

## 3. Posttranscriptional Regulation of* HLA-G*

As previously stated, there is no consensus regarding the positions of the nucleotide variation in the* HLA-G* 3′UTR, considered to be located mostly in exon 8. Since there is no official information regarding the* HLA-G* 3′UTR sequences, the nucleotide positions used in the present study follow those previously reported by our group [1, 6, 27], that is, inferring polymorphic sites in 3′UTR using the original* HLA-G* sequence described by Geraghty and colleagues [103] and considering nucleotide +1 as the Adenine of the first translated ATG (similar to the IMGT/HLA description). In the* HLA-G* 3′UTR, there is a well-studied polymorphism that consists of a 14-nucleotide deletion (rs371194629 or rs66554220), also known as the 14-bp indel (insertion/deletion) polymorphism. The sequence used as a model for the* HLA-G* promoter structure (NG_029039) does not present this 14-nucleotide sequence (that would be inserted between nucleotides +2960 and +2961). Given that the presence of these 14 nucleotides is also found in gorillas and chimpanzees, it should represent the ancestor allele, and the 14 bp sequence should be included in the 3′UTR reference sequence. Therefore, any position after nucleotide +2960 is taken considering the original NG_029039 sequence plus 14 bases. For instance, the polymorphism at the +3142 position discussed later in this review refers to the +3128 nucleotide in the NG_029039 reference sequence.

Due to a premature stop codon (positions +2536 to +2538 in NG_029039), the* HLA-G* gene presents a relatively large 3′UTR genomic sequence that extends up to the +3292 nucleotide, encompassing approximately 754 nucleotides. Inside the 3′UTR genomic region, there is an intron that is spliced out, giving rise to the mature* HLA-G* mRNA with a 3′UTR sequence of approximately 397 nucleotides (considering the presence of the 14 bases discussed earlier). This 3′UTR is a key feature for transcriptional* HLA-G* regulation, which is important for (i)* HLA-G* mRNA stability, (ii) targeting specific microRNAs [104], and (iii) polyadenylation signal in the AU-rich regulatory mRNA element [105]. The mRNA availability for translation, as well as consequent protein production and maturation, is constantly balanced by the opposing forces of transcription levels and mRNA decay. The transcription level is mainly driven by the 5' regulatory region and the presence of specific transcription factors, while mRNA decay is mainly driven by its intrinsic stability (which is dependent on the nucleotide sequence) and the action of microRNAs. MicroRNAs may negatively regulate gene expression by translation suppression, RNA degradation, or both [104, 106–108]. The first miRNA was reported in 1993 [109], and more than 2000 human microRNAs have been reported to date [110, 111].

**Figure 3:**
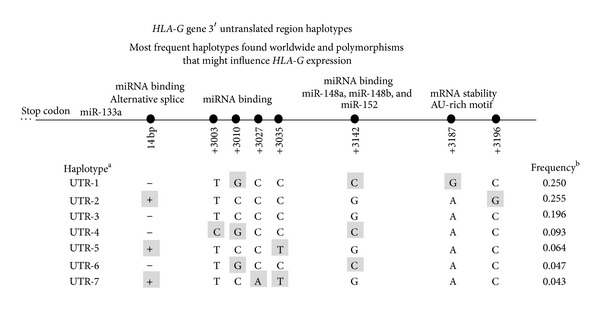
The most frequent haplotypes of the* HLA-G* 3′ untranslated region according to the 1000 Genomes data. ^a^Haplotypes were named according to [27]. ^b^Haplotype frequencies considering 21 worldwide populations [28].

The* HLA-G* 3′UTR presents several polymorphic sites, some of which have been associated with differential HLA-G expression profiles. Although the* HLA-G* 3′UTR segment is quite short compared to the same region in other genes, it presents at least eight polymorphic sites that are frequently found in worldwide populations (Figure 3). The* HLA-G* 3′UTR variability and haplotypes were systematically explored in a Southeastern Brazilian population, in which seven frequent haplotypes were described, encompassing these eight polymorphic sites, designated UTR-1 to UTR-7, and a rare one named UTR-8 [27]. The relationship between* HLA-G* 3′UTR polymorphisms (especially for the 14 bp polymorphism) and other variable sites in the* HLA-G* coding and promoter region was also previously explored [77, 78, 105, 112, 113]. Furthermore, several populations were evaluated regarding these polymorphic sites, including additional samples from other Brazilian regions and other worldwide populations, and the same pattern of 3′UTR variability has been observed [6, 27, 28, 85, 102, 114–121]. Recently, the variability at the* HLA-G* locus was explored by using the 1000 Genomes data [28, 102] and, taking together all of these studies in the last decade, it became clear that the same 3′UTR pattern observed in Brazilians [27] is found worldwide, with just some new low frequency haplotypes.

Most of the polymorphisms present in the* HLA-G* 3′UTR may influence the HLA-G expression profile by different mechanisms. Since they are present in a short mRNA sequence with just some nucleotides apart, and since the pattern of haplotypes is quite conserved [28, 102], the influence of each polymorphic site on the HLA-G expression profile may not be independent of other polymorphic sites; that is, extended haplotypes should be considered due to the cumulative effects of different polymorphisms. For example, the +3003, +3010, +3027, and +3035 polymorphic sites encompass only 32 nucleotides that are also in linkage disequilibrium with each other and in linkage disequilibrium with variable sites in the coding and promoter segments [6] (Figure 3).

The first* HLA-G* 3′UTR polymorphic site associated with HLA-G expression levels was an indel (insertion/deletion) variant known as the 14 bp polymorphism. This polymorphism is characterized by the removal of a 14-nucleotide segment [122] between positions +2961 and +2974, and it presents high frequency in all populations studied so far. The ancestor allele (the 14 bp presence or insertion) is also found in gorillas and chimpanzees [1]. The 14 bp polymorphism has been associated with the magnitude of HLA-G production [77, 123–125], modulating* HLA-G* mRNA stability [113, 126–128] and also as a target for microRNAs [106]. In general, the presence of the 14-nucleotide sequence (5′-ATTTGTTCATGCCT-3′) has been associated with lower HLA-G production for most membrane-bound and soluble isoforms in trophoblast samples [77, 78, 123, 125, 128]. However, Svendsen and colleagues observed the opposite when K562 cells were transduced with ins-14 bp HLA-G1 or with del-14 bp HLA-G1, in which the expression of HLA-G1 was found to be higher for ins-14 bp cells compared to del-14 bp cells [124]. Moreover, this 14-base sequence was also associated with an alternative splicing of the* HLA-G* transcript, in which 92 bases from the mature 3′UTR* HLA-G* mRNA were removed (including the 14-base sequence itself) [113, 128], and these smaller transcripts were reported to be more stable than the complete transcript [126]. Though influencing mRNA stability, only a fraction of the mRNA bearing these 14 nucleotides is further processed with the removal of 92 bases, and the greater stability apparently does not compensate for the lower HLA-G levels associated with the 14-base sequence. Nevertheless, there are controversial results regarding the influence of this polymorphism in HLA-G expression and alternative splicing.

The following four polymorphic sites, frequently found in the* HLA-G* 3′UTR in worldwide populations, are present at positions +3003, +3010, +3027, and +3035 [6, 27]. Although no specific regulation mechanism has been described regarding these polymorphic sites, they might influence microRNA binding [106]. Additional polymorphic sites around this small* HLA-G* 3′UTR segment are infrequently observed in worldwide populations, including the +3001 C/T polymorphism observed in Senegalese and Northeastern Brazilian populations [115, 116] and the +3033 C/G polymorphism observed among Northeastern Brazilians [115]. Although there are no studies evaluating the functional properties of these polymorphic sites, an* in silico* study reported that several microRNAs might target this small segment [106].

The nucleotide variation at position +3142 has been associated with the magnitude of HLA-G expression by posttranscription mechanisms, such as the interaction with microRNAs. It was functionally and computationally demonstrated that this variation site would influence the binding of specific microRNAs, including miR-148a, miR-148b, and miR-152 [129]. The presence of a Guanine at the +3142 position increases the affinity of this region for these microRNAs, hence decreasing* HLA-G* expression by mRNA degradation and translation suppression [106, 129, 130]. This polymorphism, together with the 14-bp polymorphism, has been considered to be the most important one regarding* HLA-G* posttranscription regulation, and methodologies have been proposed to quickly type these polymorphic sites [131, 132]. At least two studies have demonstrated that the +3142 C/G polymorphic site may influence HLA-G expression by modulating the mRNA interaction with miR-152, particularly in bronchial asthma [129, 133]. However, there is no consensus regarding the influence of this polymorphic site on the binding of such microRNAs, since another functional study did not detect this influence [134]. Instead, it was reported that both miR-148a and miR-152 downregulate HLA-G expression, irrespective of the +3142 C or G alleles [134]. These microRNAs have already been reported to modulate the expression of another classical HLA class I gene,* HLA-C* [135]. Interestingly, only HLA-C and HLA-G are usually found at the maternal-fetal interface, indicating the presence of some sort of coordinated regulation. Similarly to miR-148a, miR-148b, and miR-152, other microRNAs have the potential to bind to the* HLA-G* mRNA 3′UTR and to influence HLA-G expression. The binding ability of these microRNAs may be potentially influenced by polymorphisms observed in the* HLA-G* 3′UTR [106].

Another polymorphic site that has been associated with the magnitude of HLA-G expression is located at position +3187 A/G. This polymorphism was associated with preeclampsia in a Canadian population [136]. The mechanism underlying such association has been attributed to the proximity of this polymorphic site to an AU-rich motif that mediates mRNA degradation. Then, the presence of an Adenine instead of a Guanine at position +3187 would lead to a decreased HLA-G expression due to the increased number of Adenines in this AU-rich motif [136].

In addition to the microRNAs that might target polymorphic sequences in the* HLA-G* 3′UTR, some microRNAs would bind to nonpolymorphic sequences and modulate HLA-G expression irrespectively of the individual genetic background. However, such approach has not yet been used and only microRNAs targeting polymorphic sequences have been evaluated. Nevertheless, the microRNA miR-133a was found to target a nonpolymorphic sequence upstream to the 14-b sequence fragment, between nucleotides +2945 and +2952, downregulating HLA-G expression (Figure 3). This phenomenon was associated with the pathogenesis of recurrent spontaneous abortion [137].

Taken together, the conserved patterns of* HLA-G* 3′UTR haplotypes and the few frequent haplotypes found worldwide [102, 116] show that only one haplotype does carry all alleles that have been associated with high HLA-G expression. This haplotype, known as* HLA-G* UTR-1 [27] (14 bp Deletion/+3003 T/+3010 G/+3027 C/+3035 C/+3142 C/+3187 G/+3186 C), does not present the 14 bp sequence; that is, it presents a 14 bp deletion, which was associated with highly soluble HLA-G expression; it presents a Cytosine at position +3142 (less sensitive to specific microRNAs targeting this region), and it exhibits a Guanine at position +3187 (increased mRNA stability). Besides possessing these three polymorphic alleles associated with high HLA-G production, UTR-1 presents some other interesting features: (i) it is one of the most frequent 3′UTR haplotypes found worldwide [116], (ii) it has been described as one of the most recent* HLA-G* 3′UTR haplotypes among the frequent ones due to its exclusive association with the presence of an Alu element that is close to* HLA-G* (20 Kb downstream the 3′UTR) [102], and (iii) UTR-1 was recently associated with higher HLA-G expression [138].

Several studies have reported that the* HLA-G* 3′UTR segment is also under selective pressures, whereby balancing selection is maintaining high levels of heterozygosis in this region [6, 27, 28, 101, 139, 140]. As observed worldwide [27, 28, 102], the two most frequent* HLA-G* 3′UTR haplotypes (UTR-1 and UTR-2) are also the most divergent ones (Figure 3). They differ in all known variable sites that might influence HLA-G expression. Therefore, the same phenomenon observed for the promoter region is also seen in the 3′UTR, in which high heterozygosis is observed between high- and low-expression haplotypes. Moreover, the rate of recombination in the* HLA-G* locus is quite low, and the pattern of linkage disequilibrium found in the* HLA-G* locus encompasses the promoter region, the coding region, the 3′UTR, and at least 20 kB downstream of the 3′UTR [102]. Thus, in general, only few frequent extended haplotypes do exist and a specific promoter haplotype is usually accompanied by the same* HLA-G* coding sequence and the same 3′UTR haplotype [6–8, 28, 77, 78, 102]. The UTR-1 haplotype, for example, is usually associated with the coding sequence for the HLA-G*01:01:01:01 allele and the PROMO-G010101a promoter haplotype [6–8, 28, 102]. Therefore, the influence of each variable site at the HLA-G transcriptional level must be considered.

## 4. *HLA-G* Coding Region Polymorphisms Influencing HLA-G Expression

The HLA-G genetic structure resembles the class I structure, in which the first translated exon encodes the peptide signal, the second, third, and fourth ones encode the extracellular *α*1, *α*2, and *α*3 domains, respectively, and the fifth and sixth ones encode the transmembrane and the cytoplasmic domain of the heavy chain. Considering the* HLA-G* coding region (from the first translated ATG to the stop codon), at least 75 single nucleotide polymorphisms (SNP) have been observed, defining the 50 currently described* HLA-G* alleles, encoding only 16 distinct proteins (IMGT, database 3.14.0, November 2013). Similarly to what has been described for other genes such as* IRF4, MYC, IFNG, *and others [141–146], it is possible that intronic or exonic nucleotide sequences may exhibit affinity for transcription factors, thereby regulating the expression of the gene; however, this subject has not yet been studied in the context of the* HLA-G* gene.

The presence of certain polymorphic sites in the coding region may also regulate the expression of the seven described HLA-G isoforms generated by alternative splicing of the primary transcript. Four of the HLA-G isoforms are membrane-bound (HLA-G1, G2, G3, and G4) and 3 are soluble (G5, G6, and G7) ones. HLA-G1 is the complete isoform exhibiting a structure similar to that of the membrane-bound classical HLA molecule, associated with *β*2-microglobulin, HLA-G2 has no *α*2 domain, HLA-G3 presents no *α*2 and *α*3 domains, and HLA-G4 has no *α*3 domain. The soluble HLA-G5 and HLA-G6 isoforms present the same extracellular domains of HLA-G1 and HLA-G2, respectively, and the HLA-G7 isoform has only the *α*1 domain [147–149]. In contrast to most of the currently described* HLA-G* alleles that may produce all membrane-bound and soluble isoforms, the presence of stop codons in the coding region may yield truncated or missing HLA-G isoforms. The HLA-G*01:05N null allele presents a Cytosine deletion in the last nucleotide of codon 129 or in the first nucleotide of codon 130 (exon 3), leading to a TGA stop signal in codon 189, yielding incomplete formation of the HLA-G1, -G4, and -G5 isoforms and normal expression of HLA-G2, -G3, and -G7 [1, 150, 151]. Similarly, the HLA-G*01:13N allele presents a C → T transition in the first base of codon 54 (*α*1 domain), yielding the formation of a premature TAG stop codon, preventing the production of all membrane-bound and soluble isoforms, and therefore it is probably not expressed [1, 152, 153].

Humans bearing allele G*01:05N in homozygosis have been reported [154–157], a fact that may indicate that soluble HLA-G molecules or molecules lacking the *α*3 domain are sufficient for HLA-G function. The frequency of the G*01:05N allele varies among different populations [1], ranging from complete absence in Amerindian populations from the Amazon, Mayans from Guatemala, and Uros from Peru [139, 151, 158], to intermediate frequencies in Africa [155] and higher than 15% in some populations of India [159], while allele G*01:13N is quite rare [152, 153]. It has been proposed that high G*01:05N frequencies are associated with high pathogen load regions, and intrauterine pathogens would act as selective agents, with increased survival of G*01:05N heterozygous fetuses. In this case, the reduced HLA-G1 expression may result in an improved intrauterine defense against infections [139, 151, 154, 160]. To the best of our knowledge, no homozygous G*01:13N has been described.

## 5. Concluding Remarks

Due to the important role of HLA-G in the regulation of the immune response and its relevant function during the course of pregnancy, the overall structure of the molecule has been maintained during the evolution process, preserving major HLA-G binding sites to leukocyte receptors and HLA-G dimer formation. On the other hand, several variable sites have been observed along the* HLA-G* regulatory regions. Although a perfunctory analysis of the many variable sites observed in the promoter region of several worldwide populations indicates that some known transcription factor target regions have also been conserved, one cannot rule out the influence of the differential action of distinct transcription factors according to promoter region variability. In contrast, most of the variable sites found in the* HLA-G* 3′UTR might influence HLA-G expression by facilitating or hindering microRNA binding and/or influencing mRNA stability.
